# Special Issue “Precision Medicine in Neurodevelopmental Disorders: Personalized Characterization of Autism from Molecules to Behavior”

**DOI:** 10.3390/jpm12060918

**Published:** 2022-06-01

**Authors:** Elizabeth B. Torres

**Affiliations:** 1 Department of Psychology, Rutgers the State University of New Jersey, Piscataway, NJ 08854, USA; ebtorres@psych.rutgers.edu; 2 Center for Biomedicine Imaging and Modeling, Computer Science Department, Rutgers University, Piscataway, NJ 08854, USA; 3 Center for Cognitive Science, Rutgers University, Piscataway, NJ 08854, USA

The Precision Medicine (PM) platform [1] has emerged as an important transformative model to help advance personalized medicine and transform translational research and clinical practices. In PM, multiple layers of the knowledge network are interconnected (Figure 1) to integrate the patient’s clinical information derived from a multitude of tests, with different levels of objectivity and subjectivity, into targeted treatments that rely on specific signatures of the patient’s health, in relation to population signatures.

The present Special Issue, entitled “Precision Medicine in Neurodevelopmental Disorders: Personalized Characterization of Autism from Molecules to Behavior”, brings together researchers from multiple disciplines to address research at various layers of the knowledge network and offer contemporary solutions to the implementation of some of the current research in autism and other neurodevelopmental disorders.

Cristina Panisi, et al. [2] discussed “Autism Spectrum Disorder from the Womb to Adulthood: Suggestions for a Paradigm Shift”, offering new avenues of inquiry and integration of research across different stages of neurodevelopment. In their manuscript, the authors highlight the need to move towards a more fluid, dynamic conception of autism, one that integrates genetics, environment, and epigenetics in a holistic manner, taking into consideration individual systemic variations, rather than doing so linearly and in a piecemeal fashion. They discuss the embryo–fetal period and the first two years of life (so-called ‘First 1000 Days’), as a critical window to detect differences at its earliest point and intervene accordingly. The work invites possible interventions while considering immune activation, gut dysbiosis, and mitochondrial impairment/oxidative stress affecting neurodevelopment during pregnancy and undermining the health of autistic individuals throughout life. The review argues for intervention at the molecular levels during early embryonic stages of neural development, as a path forward that is now realizable thanks to recent advances in omics. A comprehensive and exhaustive pathogenic research approach to autism is advanced in this paper, as an actionable medical resource that can go beyond theoretical ideas, into practical implementation. This work offers a bird’s view of this lifelong condition while integrating these multiple layers of knowledge, while the papers below address relevant issues at each one of the individual layers of the knowledge network.

At the important level of patient’s self-reports and observational behavioral inventories, encompassing the perception of autistic individuals by others, we find the manuscript by Demiy, et al. [3], entitled “A Child’s Perception of Their Developmental Difficulties in Relation to Their Adult Assessment: Analysis of the INPP Questionnaire”. In this paper, the authors compare the perception of teachers, parents, and clinicians to those of the child, using clinical questionnaires from the Institute for Neuro-Psychological Psychology (INPP). The questions focus primarily on psychomotor problems related to balance, motor coordination and concentration, as well as school skills. The work reports that children self-perceived these issues significantly stronger than their parents did, and educators and therapists differed from the parents’ opinion, particularly in matters related to attention and concentration. The results highlight the important amount of information that escapes the naked eye of the observer. They underscore the need to consider the internal states of the autistic person, as expressed by the autistic individuals. The study concludes that “Children perceive their difficulties much more seriously than adults.” and suggests that “Talking and the support of adults can make it easier for a child to overcome developmental difficulties.”

Insights into automated behavioral assessments at the intersection of clinical reports and the layer of behaviors are offered by Cavus, et al. [4], in their paper, entitled “A Systematic Literature Review on the Application of Machine-Learning Models in Behavioral Assessment of Autism Spectrum Disorder”. The authors address the critical need for computationally driven assessments in an exponentially growing ASD phenomenon that requires, but lacks, trained diagnosticians with high scoring reliability. Despite good evaluation metrics achieved by the ML models, there remains scarce evidence on their readiness for clinical implementation. The review highlights numerous challenges associated with data-centric techniques and their misalignment with the conceptual basis upon which professionals diagnose ASD. Their systematic review proposes vital considerations for real-life implementation of ML-based ASD screening and diagnostic systems that other authors in the Special Issue take on.

The work by Washington, et al. [5], entitled “Precision Telemedicine through Crowdsourced Machine Learning: Testing Variability of Crowd Workers for Video-Based Autism Feature Recognition”, provides a clear example of implementation of ML methods and innovative approaches to diagnosing ASD. The results from their work demonstrate that while the crowd can produce accurate diagnoses, there are intrinsic differences in crowdworker ability to rate behavioral features. The authors propose a novel strategy for the recruitment of crowdsourced workers, to ensure high-quality diagnostic evaluations of autism, and potentially many other pediatric behavioral health conditions. This work represents a viable step in the direction of crowd-based approaches for more scalable and affordable precision medicine.

Moving along to the layer of behavioral analyses, Ryu, et al. [6] offer new methods to assess pain in their paper, entitled “Personalized Biometrics of Physical Pain Agree with Psychophysics by Participants with Sensory over Responsivity”. This work underscores the importance of combining digital data from biosensors with clinical inventories, to produce clinically interpretable digital biomarkers of pain. Using EEG activity and new analytical methods, the researchers from a multitude of fields collaboratively joined efforts with clinicians to study sensory issues commonly found in autism. The group characterizes pain by standardizing the moment-by-moment fluctuations in biophysical signals derived from EEG brain activity. These signals from the central nervous systems (CNS) reflect the person’s experience of temperature-based stimulation at the periphery. A type of gross data that is often disregarded as noise by traditional analytic methods, here, precisely characterizes the lingering sensation of discomfort raising to the level of pain, individually, for each participant. The work shows fundamental differences between the SOR group in relation to controls and provides an objective account of pain that is congruent with the subjective self-reported data. This integrative approach offers the potential to build a standardized scale useful to profile pain levels in a personalized manner across the general population.

Within the layer of behavioral assessments, another paper by Ryu and Torres [7], entitled “The Autonomic Nervous System Differentiates between Levels of Motor Intent and End Effector”, examines differences in volitional control that are capturable in personalized form, through the person’s fluctuations in heart-rate variability. The work alludes to the potential to bridging motor control and cognitive science by tracking peripheral activity as reafferent input that is convolved with micro-motor (kinesthetic) reafference, harnessed from continuous streams of interleaved intentional and spontaneous movements. Using new analytics that do not discard the so-called “gross data” as noise, the authors find that when the action is intended, the heart signal from the Autonomic Nervous Systems (ANS) leads the body kinematics signals. In stark contrast, when the action segment spontaneously occurs without instructions, the heart signal lags the bodily kinematics signals. They conclude that the ANS can differentiate levels of intent, a result that has translational value, and actionable scalability, given the ubiquitous presence of commercially available, off-the-shelf wearable biosensors embedded in smart watches and phones. These biosensors reliably register heart-rate variability in natural situations, as the person, e.g., wears a smart watch and checks the outputs in an app. In this sense, using such signals combined with the analytics reported in the methods, it becomes feasible to study and infer moment-by-moment cognitive states from motor and autonomic data streams.

Finally, a path forward integrating these disparate layers of knowledge is offered by Torres in their paper, entitled “Reframing Psychiatry for Precision Medicine” [8], with a direct application to autism in a paper, entitled “Precision Autism: Genomic Stratification of Disorders Making Up the Broad Spectrum May Demystify Its Epidemic Rates” [9]. These two papers lay out a possible way to help cope with the heterogeneous nature of developmental disorders on a spectrum by leveraging the genomics revolution and automatically stratifying the disorder into subgroups with common genetic pools, according to genes’ expressions on fundamental tissues underlying all social behaviors that define the disorders in the first place. Combining digitized behaviors (naturally and unobtrusively attainable during clinical tests), with observational data informed by clinical criteria, and integrating genomic information, will help treat such disorders in autism by leveraging advances from different fields. This approach could significantly help improve a person’s quality of life and redirect resources differently towards fields that offer real solutions for everyday independent living. Further advancing research questions in autism informed by the person’s self-reports on internal states is also possible under a new statistical platform that harnesses individual variations present in the continuous stream of data that we collect at each of these layers of the knowledge network in Figure 1. From variations in genes’ expression to variations in genes’ network interactions, to variations in fluctuations of biophysical rhythms registered from the CNS, the PNS and the ANS, we can help integrate these disparate layers of information under a common computational framework that does not throw away data and relies on a systemic approach, integrating information from the human body and brain, as they dynamically change over time.

This Special Issue provides an example of interdisciplinary collaboration occurring today at multiple levels of inquiry across complex, nonlinear dynamics, and the stochastic variations that continuous streams of data offer to contemporary medicine. A new unifying model that helps us integrate such information and track it over time has already made its presence visible in our labs, clinics, and homes. Indeed, we are at an inflection point in medicine [1], poised for a clinical revolution that has leveraged neuroscience, genomics, and the wearable biosensor revolution of the last decade. Personalized Medicine has already arrived.

## Figures and Tables

**Figure 1:**
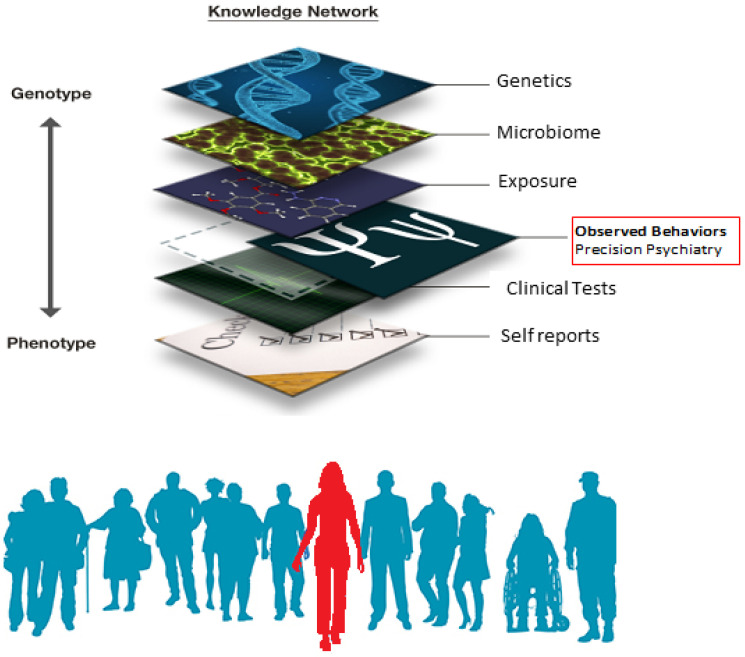
The Precision Medicine platform applied to the fields of Psychiatry and Psychology, can integrate information from several layers of the knowledge network, to help develop targeted behavioral and genomic personalized treatments to improve mental health. The layer of observed behaviors can now be digitized thanks to the wearable sensors revolution and integrated with clinical criteria and self-reports, to provide interpretable digital biomarkers for Precision Psychiatry (Figure courtesy of Dr. C.P. Whyatt and the Rutgers University Sensory Motor Integration lab).
